# Empathy Is a Protective Factor of Burnout in Physicians: New Neuro-Phenomenological Hypotheses Regarding Empathy and Sympathy in Care Relationship

**DOI:** 10.3389/fpsyg.2016.00763

**Published:** 2016-05-26

**Authors:** Bérangère Thirioux, François Birault, Nematollah Jaafari

**Affiliations:** ^1^Unité de Recherche Clinique Intersectorielle en Psychiatrie à Vocation Régionale Pierre Deniker, Centre Hospitalier Henri LaboritPoitiers, France; ^2^Faculté de Médecine et de Pharmacie, Département de Médecine Générale, Université de PoitiersPoitiers, France; ^3^Institut National de la Santé et de la Recherche Médicale CIC-P 1402 du Centre Hospitalo-Universitaire de Poitiers, Institut National de la Santé et de la Recherche Médicale U 1084, Experimental and Clinical Neuroscience Laboratory, Groupement de Recherche Centre National de la Recherche Scientifique 3557, Université de PoitiersPoitiers, France

**Keywords:** burnout, physicians, empathy, sympathy, care relationships, self-other distinction, prevention, self-regulation

## Abstract

Burnout is a multidimensional work-related syndrome that is characterized by emotional exhaustion, depersonalization—or cynicism—and diminution of personal accomplishment. Burnout particularly affects physicians. In medicine as well as other professions, burnout occurrence depends on personal, developmental-psychodynamic, professional, and environmental factors. Recently, it has been proposed to specifically define burnout in physicians as “pathology of care relationship.” That is, burnout would arise, among the above-mentioned factors, from the specificity of the care relationship as it develops between the physician and the patient. Accordingly, experimental studies and theoretical approaches have suggested that burnout and empathy, which is one of the most important skills in physicians, are closely linked. However, the nature of the relation between burnout and empathy remains not yet understood, as reflected in the variety of theoretical and contradictory hypotheses attempting to causally relate these two phenomena. Firstly, we here question the epistemological problem concerning the modality of the burnout-empathy link. Secondly, we hypothesize that considering the multidimensional features of both burnout and empathy, on one hand, and on the other hand, the distinction between empathy and sympathy enables to overcome these contradictions and, consequently, gives a better understanding of the relationship between burnout and empathy in physicians. Thirdly, we propose that clarifying the link between burnout, empathy and sympathy would enable developing specific training in medical students and continuous professional formation in senior physicians and would potentially contribute to the prevention of burnout in medical care.

## Introduction

Professional exhaustion syndrome—or “burnout”—in medical care is higher than in other professions (Shanafelt et al., 2012). Burnout in general, i.e., irrespective of profession, has a multifactorial origin. In fact, a combination of personal, developmental-psychodynamic, professional, and environmental factors would provoke burnout (Truchot, 2006). However, an additional factor has been recently advanced to be peculiar to burnout in physicians, i.e., the relationship toward the *other* as *patient*. Therefore, burnout has been defined as “pathology of care relationship” (Galam, 2007). It means that there would be a potential link between the weakening of the Self as it occurs in burnout and the specificity of the relationship to others *as patients* in medical care. Burnout would arise, thus, from this dynamic and care-specific relationship between the Self (physician) and the other (patient). Concordant with this view, experimental studies and theoretical approaches suggest that burnout and empathy are closely linked (Gleichgerrcht and Decety, 2013; Lamothe et al., 2014; Tei et al., 2014).

However, the nature of the relationship between burnout and empathy in physicians is not yet understood. This is reflected in the large variety of theoretical hypotheses that try to explain the causal relation between burnout occurrence and empathy, and in different levels of contradiction opposing these same hypotheses. As an example, empathy has been assumed to cause (Figley, 2002; Nielsen and Tulinius, 2009) and, inversely, to prevent burnout (Halpern, 2003). Burnout has also been proposed to alter empathy (Shanafelt et al., 2005; Brazeau et al., 2010; Zenasni et al., 2012). If there is a form of consensus regarding the link between burnout and empathy, the contradictory nature of these explanatory hypotheses raises the epistemological problem of the modality of this link.

We propose that taking into account the multidimensional aspect of both burnout and empathy, on one hand, and on the other hand, the distinction between empathy and sympathy, enables to overcome these contradictions and, consequently, gives a better understanding of the relationship between burnout and empathy in physicians. Moreover, we believe that understanding the modality of this link is important to contribute to the prevention of burnout in developing empathy-based training for medicine students and continuous formation for senior physicians.

## Burnout in care relationship

The syndrome of professional exhaustion was described for the first time in 1959 by C. Veil (Veil, 1959) whilst the term of “burnout” was later introduced by H. J. Freudenberger in 1974 (Freudenberger, 1974). Initially, burnout has been defined as a syndrome affecting individuals who are professionally engaged with others. Since the nineties, this definition has been extended to all individuals who are psychologically engaged in their profession. There is no diagnostic classification of burnout in ICD-10 or DSM-V.

Burnout consequences are deleterious within the physician-patient relationship. It first alters the physicians' well-being and health and is characterized by a diminution of the quality of life and occurrence of organic and psychological pathologies (Galam et al., 2013). It also alters the quality of care with serious repercussions on the patients' health and is associated with an increase of negligence and medical errors (Shanafelt et al., 2002; Reader and Gillepsie, 2013). In addition to its respectively direct and indirect effects on physicians' and patients' health, burnout has a non-negligible cost for the society, leading to a significant augmentation of absenteeism, career changes, and, modifications in care offers and distributions (Lichtenstein, 1984; Williams et al., 2010).

Burnout symptoms are varied and non-specific. These can be physical (abdominal and/or musculoskeletal pains, asthenia), psychical (depressive and anxious disorders potentially leading to suicide), psycho-behavioral (sleep disorders, hyperactivity, modification of life hygiene, augmentation of addictive behaviors such as tobacco or alcohol consummation) or cognitive (negative perception and attitudes regarding family members) (Dyrbye et al., 2005, 2006; West et al., 2006; Pejušković et al., 2011). Moreover, burnout is a multidimensional syndrome that is defined by three successive or co-existing dimensions. Emotional exhaustion [EE] is a feeling of emptiness, emotional saturation as well as physical and psychical fatigue, generating difficulties to perform usual professional activities. Depersonalization [DEP]—or cynicism—consists of negative, distant and/or impersonal attitudes toward others, leading to isolation and rejection. Diminution of personal accomplishment [PA] is reflected in a self-esteem decrease, self-depreciation, feeling of failure and culpability. Burnout severity in general and relative to each of its three dimensions is evaluated in physicians by means of the 22-items Malash Burn Out Inventory [MBI] (Maslach and Jackson, 1981).

Burnout prevalence through the different care professions (i.e., midwifes, physicians, nurses, surgeons etc.) has been largely investigated and documented. Depending on care professions and countries, burnout concerns 50% of healthcare professionals. Especially, 65% of European general practitioners (GPs) (Soler et al., 2008) indeed suffer from burnout. Burnout prevalence linked to emotional exhaustion, depersonalization and diminution of personal accomplishment is respectively about 43, 35, and 32% in Europeans GPs. Burnout also affects medical students. It has been shown that 58% of French GPs in their residency training report burnout symptoms. Specifically, 12% of them suffer from high scores to the MBI emotional exhaustion subscale [MBI EE], 34% from high scores to the depersonalization subscale [MBI DEP] while 39% have low scores to the personal accomplishment subscale [MBI PA] (Galam et al., 2013). More importantly, 7% of GPs residents have extremely high scores to the MBI EE and MBI DEP associated with extremely low scores to the MBI AP, i.e., scores which are therefore considered as pathological. Comparable data have been reported in residents in urology, gynecology and anaesthesia. It has been further shown that depersonalization increases correlatively with the number of years of medical education (Hojat et al., 2004; Handford et al., 2013). More precisely, burnout is significantly high in the 1st year of medical education, decreases in the 2 following years but increases progressively and intensively from the 4th until the 6th year i.e., when students prepare their residency exam (Truchot, 2006).

Burnout etiopathogeny in physicians and medical students is still debated. Personal, developmental-psychodynamic, professional and environmental factors have been advanced to increase burnout risks. Particularly, unbalance between one's professional and private life but also between self- and other-oriented interests are important burnout risk factors (Truchot, 2006) (Table 1). As an example, MBI scores increase with a too high workload and lack of recognition but decrease when more time is given to one's private life (Truchot, 2006). As mentioned above, it has been recently proposed to consider burnout as pathology of care relationship (Galam et al., 2013) and to relate burnout occurrence to empathy disorders in physicians. This hypothesis is particularly interesting as medical teachers, faced with this burnout increase in their students, are currently addressing the issue that burnout occurrence interacts with how clinical and professional competences are taught. They specifically focus on the question of empathy because empathy is acknowledged in the medical education as one of the most indispensable skills in physicians (Medical Schools Objectives Writing Group, 1999; General Medical Council, 2009).

**Table 1:** **Preventive and risk factors of burnout in physicians**.

	**Risk**	**Preventive**
Professional factors	High workload	Balanced workload
	Low support from hierarchy and colleagues	High support from hierarchy and colleagues
	Lack of room for maneuver	Room for maneuver
	Lack of work recognition	High work recognition
	High emotional exigencies related to work	Balanced emotional exigencies related to work
	Lack of equity	Satisfying level of equity
	Conflict between the professional values of the institution and one's own values	Accordance between the professional values of the institution and one's own values
	Exaggerated professional engagement	Balanced professional engagement
Personal factors	Not enough time given to private life	Time given to private life
	Substances consummation and abuse (alcohol, drugs …)	Healthy lifestyle without substances abuse Sport practice
	Not enough use of recreation, hobbies, exercise promoting life-work balance	Satisfying use of recreation, hobbies and exercise promoting life-work balance
	Male physicians are more prone to suffer from depersonalization Female physicians are more prone to suffer from emotional exhaustion	
	Young physicians « midlife » crisis (aged from 45 to 55)	
	Perfectionism Competitiveness Personality type: nevrosism	Personality type: extraversion
	Difficulties to use copying strategies centered on specific encountered problems and emotions	Facilities to use copying strategies centered on specific encountered problems and emotions Mindfullness practice
Environmental factors	Low support from family and friends	High support from family and friends
	Single status	Marital status
	No children or children younger than 6 years old	Children older than 6 years old
Care-relationship factors	Sympathy toward patients Emotional contagion Self-other confusion	Empathy toward patients Emotional regulation Self-other distinction

Table indicates the professional, personal, environmental and care-relationship factors that prevent or provoke burnout in physicians.

## Neuro-phenomenology of empathy and sympathy

In social-cognitive neurosciences, empathy is frequently defined as the capacity to feel and share the emotions of others. Therefore, empathy is described as an affective state and emotional reactions that are caused by emotional sharing. Empathy would be further associated with the subjective awareness that these emotions originate in another individual and to a *minimal* self-other distinction (Hein and Singer, 2008; Decety and Michalska, 2009). Although, emotions are the core of empathy, this definition is not totally satisfying. Firstly, it reduces empathy to a strict emotional dimension. However, according to the traditional phenomenological theories that have introduced the term and concept of empathy (Lipps, 1913; Vischer, 1927; Husserl, Hua XII–XV), empathy is a feeling that enables to access the embodied mind of others “in their bodily and behavioral expressions (Zahavi, 2008)—irrespective of the content (emotions, sensations, actions etc. …) of the others' lived experience. Secondly, this definition gives a minor role to the vestibular mechanisms and higher-order cognitive functions that underpin self-other distinction. Thirdly, it tends to conflate empathy and sympathy. Although sharing basic processes (feelings and autonomic processes) and outcomes (moral development and prosocial behavior) (Hojat et al., 2011a,b; see also Decety and Michalska, 2009; Walter, 2012), empathy and sympathy are both distinguished on phenomenological and neuro-functional levels.

### Phenomenological distinction between empathy and sympathy

Empathy and sympathy respectively consist of “feeling *into*” and “feeling *with*” someone else (from the German *ein* [into] vs. *mit* [with]—*fühlen* [to feel]) (Jorland and Thirioux, 2008; Gelhaus, 2011; Hojat et al., 2011b; Thirioux et al., 2014). This feeling refers to the mental experience of one's physiological and bodily states and changes (Damasio and Carvalho, 2013) that are triggered by the perception of the others' motor, somatosensory, emotional, affective or intentional lived experience. In contrast, the different spatial meaning of their prefixes (*ein* [into] and *mit* [with]) indicates how empathy and sympathy distinguish on a phenomenological level (Thirioux et al., 2014). This distinction relies upon three key phenomenological components of bodily self-consciousness: self-identification (the experience of owning a body), self-location (the experience of where I am in space) and first-person (egocentered) perspective (the experience from where I perceive the world) (Blanke, 2012). These components are essential in self-other relationships. In fact, the spatial determination of one's physical body lies at the basis of self-other distinction: I am physically *here* (*hic*) but the other is physically *there* (*illic*). Empathy and sympathy differentially modulate self-location and first-person perspective (Thirioux, 2011; Thirioux et al., 2014).

**Figure 1:**
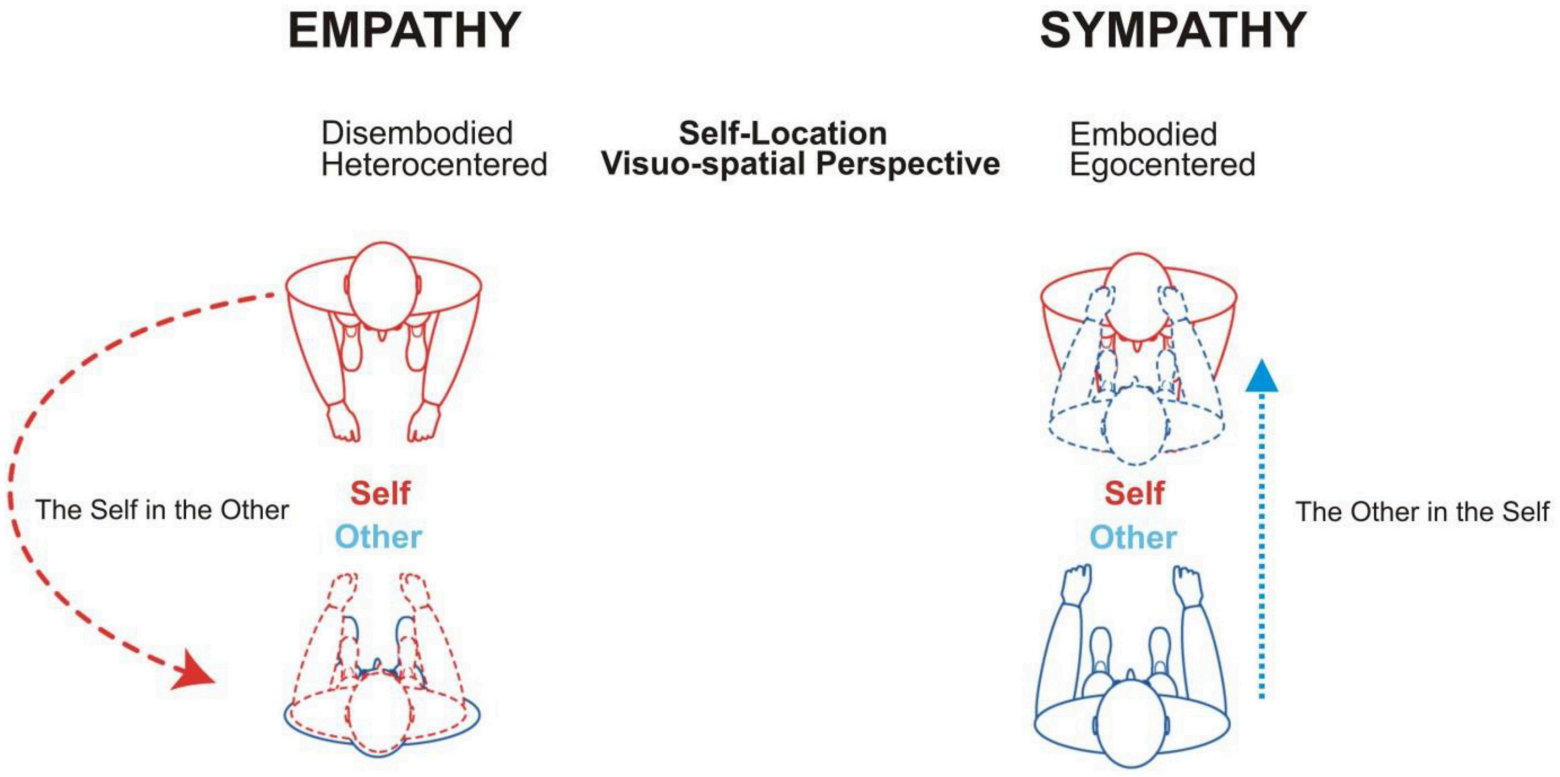
**Theoretical schema of the phenomenological distinction between empathy and sympathy**. Empathy (feeling *into*) and sympathy (feeling *with*) are associated with distinct self-location (disembodied vs. embodied) and visuo-spatial (heterocentered vs. egocentered) mechanisms (adapted from Thirioux et al., 2009, 2010).

Empathy refers to the capacity to mentally decentre oneself into others (feeling *into* someone else). The awareness of being outside the other person and having to reach him/her (Gelhaus, 2011) is the prerequisite for empathy. It requires an own-body mental imagery and spatial transformation process consisting in a translocation of one's egocentered perspective in the other's body. That is, in mentally centring one's own-body axis onto the other's body axis in a rotation-like manner. Accordingly, individuals imagine themselves to be located in the other's body and experience the world from his/her body position (Thirioux et al., 2009, 2010, 2014) (Figure 1). However, imagining oneself to be located in the body position *of someone else* necessitates that this other person still appears to me as other *than* me, i.e., it necessitates the parallel awareness to be physically located at a given place in space that is different from that of the other person (Berthoz, 2004; Thirioux, 2011). Consequently, empathy encompasses three core components: disembodied self-location (enabling to mentally put oneself into the other's body), heterocentered (Degos et al., 1997) visuo-spatial perspective-taking (coding for the others' visuo-spatial perspective) and parallel coding of one's body position in space (egocentered). This threefold process enables feeling, thinking and understanding what the other *as other*—i.e., precisely as the other is not me—is feeling and thinking from his/her own viewpoint and lived experience. As such, feeling into someone else requires a clear consciousness of one's ipseity so that the other appears in his/her alterity (Gelhaus, 2011; Thirioux, 2011). When empathizing, individuals are neither losing themselves in others nor merging with others. Empathy relies upon a conscious and not only minimal distinction between viewpoints (mine vs. his/hers) and current lived experiences (mine vs. his/hers) and, therefore, maintains self-other distinction. In summary, empathy is the capacity to feel and understand the emotional, affective but also motor, somatosensory, or intentional experience of others and their associated mental state, while adopting the others' visuo-spatial perspective and psychological viewpoint and consciously maintaining self-other distinction (Thirioux et al., 2014).

In contrast, when sympathizing, individuals are feeling the same thing as others are feeling (the same kind of inner state, Gelhaus, 2011) and at the same time (Olinick, 1987), tending to merge identities (Wilmer, 1968). This identification between self and others is prompted by the attribution of the other's experience to oneself as if individuals were the other person (Gelhaus, 2011). This self-attribution is based upon a specific own-body mental imagery and spatial transformation process in which individuals are mapping the other's body into their own-body and reducing the other's perspective to their egocentered perspective. That is, in sympathy individuals are centring the other's body axis onto their own-body axis in a mirror-like manner (Figure 1). Sympathy relies, thus, upon both embodied self-location and egocentered visuo-spatial perspective and consists in attributing to oneself what other individuals are currently experiencing (Thirioux et al., 2014). The usual definition of empathy, according to which empathy consists in sharing the emotions of others, rather refers to sympathy than empathy.

### Neurophysiology of empathy and sympathy

As multidimensional phenomenon (Davis, 1994; Preston and de Waal, 2002; Preston, 2007; Thakkar et al., 2009), empathy relies upon the cooperation of automatic, emotional, cognitive, visuo-spatial, and self-regulation processes. On a neuro-functional level, this complex cooperation of processes is underpinned by the integration and modulation of specific activations in topographically distributed and functionally distinct brain networks (Berthoz, 2004; Decety and Jackson, 2004; Decety, 2007).

The automatic components of empathy refer to first-person-like simulation processes in which individuals internally but partially reproduce another person's subjective experience (Goldman, 2006). Mirror neuron system (MNS) (di Pellegrino et al., 1992) has been advanced as the most plausible neurobiological basis for simulation (Gallese et al., 1996; Gallese, 2001; Fabbri-Destro and Rizzolatti, 2008). It is now well-documented through numerous functional magnetic resonance imaging (fMRI) and electrical neuroimaging (EEG) studies that action execution and observation trigger isomorphic activations in the motor system, i.e., in the inferior frontal gyrus (IFG), inferior parietal lobule (IPL) premotor cortex, and superior temporal sulcus (STS) (Iacoboni et al., 1999; Buccino et al., 2001; Grèzes et al., 2003; Binkofski and Buccino, 2006; Newman-Norlund et al., 2007). Similarly, comparable functional isomorphism has been reported in the anterior part of the bilateral insula, rostral part of the anterior cingulate cortex, cerebellum and brainstem when people observe the emotions of someone else (e.g., disgust) and when they are feeling the same emotions (Wicker et al., 2003). Putative MNS functioning has been also described in the secondary somatosensory cortex when individuals are touched (e.g., on their leg) and observe another person being touched (e.g., on his/her leg) (Keysers et al., 2004).

The cognitive components of empathy refer to controlled second-person-like processes whereby individuals represent and understand the mental state of others. These Theory of Mind- (ToM) (Premack and Woodruff, 1978) or mentalizing-based processes (Frith and Frith, 2003) enable representing and attributing mental states to oneself and others by means of logical inferences, contextual information and psychological perspective-taking (Walter, 2012). This generates specific activations in the ventro/dorsomedian prefrontal cortex (vmPFC / dmPFC), temporo-parietal junction (TPJ), anterior STS (aSTS), precuneus and temporal poles (TP) (mentalizing network [MENT]). Within the MENT, the left TPJ encodes the visuo-spatial perspective of others and perspective ownership (Thirioux et al., 2010; McCleery et al., 2011) whilst the vmPFC calculates the others' psychological viewpoint (Frith and Frith, 2003, 2006a,b).

Visuo-spatial processes in empathy enable to decentre the self into the other and to switch from an egocentered to a heterocentered referencing system. This visuo-spatial shift, that enables adopting another person's psychological perspective, is further associated with a dynamic coding of one's own-body and egocentered position. This twofold ego-heterocentered coding maintains the distinction between self and others. It avoids inappropriately attributing to oneself the observed experience as lived by others. These visuo-spatial mechanisms are underpinned by specific activations in the vestibular system, i.e., in the right TPJ (egocentered coding), left TPJ (heterocentered coding) and insula (Thirioux et al., 2014).

Finally, empathy relies upon self-regulation and inhibitory components. These are sustained by the right dorsolateral PFC (dlPFC) within the executive system (Decety et al., 2010; McCleery et al., 2011; Thirioux et al., 2014). On an emotional level, self-regulation mechanisms enable inhibiting negative emotions that are spontaneously generated when observing the others' distress (Decety and Moriguchi, 2007; Decety et al., 2008). On a visuo-spatial level, these enable partially inhibiting one's egocentered visuo-spatial perspective. These are, thus, necessary for the dynamic ego-heterocentered referential coding on which the self-other distinction is based. Accordingly, by means of a partial top-down control of one's egocentered perspective, these neo-cortical self-regulation mechanisms inhibit the attribution to oneself of what others are experiencing. These also contribute to monitor the tendency to project onto others what individuals would have probably felt in a similar situation.

The expression “to know how it feels like to” that was introduced by Nagel to describe empathy perfectly accounts for the integration of these cognitive (“to know”) and feeling-related (“to feel”) components of empathy (Nagel, 1974).

Recent fMRI and EEG data tend to validate the integrative approach of empathy. These show that empathy relies upon the functional integration of activations within the MNS, MENT, executive and limbic networks (Schulte-Rüther et al., 2007; Schippers et al., 2010; Anders et al., 2011; Schnell et al., 2011; Ciaramidaro et al., 2013). As an example, observing the emotions of someone experiencing ostracism generates activations in the anterior (aSTS, vmPFC) and posterior (PT, TPJ) MENT regions in association with mirroring activations in the hippocampus and amygdala (Schnell et al., 2011). This simultaneous top-down neocortical and bottom-up limbic activity suggests that observing another individual's emotions relies upon mentalizing about the other's emotional and affective state, on one hand, and, on the other hand, upon the use and partial reactivation of stored information relative to one's own emotional past experiences (hippocampus / autobiographical memory; amygdala /emotional processing). Studies further demonstrated a bidirectional functional connectivity—i.e., a reciprocal causal relationship—between activations in specific MNS, MENT and limbic areas when observing decision making in emotional and moral dilemma contexts (Raz et al., 2014).

Using cortical dynamics analyses, recent EEG studies on self-other motor interaction have shown that empathy and sympathy differentially modulate the functional integration of the MNS, MENT and self-regulation networks (Thirioux et al., 2010, 2014). Between ~60 and ~330 ms post stimulus onset (PSO), both empathy and sympathy generated MNS activations that progressed from the right occipital cortex to the IPL *via* the middle temporal gyrus (MTG) and STS. At ~330–440 ms, although empathy and sympathy commonly activated the right IFG, empathy but not sympathy triggered specific activations in the right dlPFC. From ~520 to ~630 ms, empathy and sympathy were respectively sustained by activations in the left TPJ and precuneus (MENT) and right premotor and secondary somatosensory cortices (MNS). This suggests therefore that sympathy, triggering the typical sequence of MNS activations, probably generated self-attribution of actions and experience sharing. In contrast, co-activations in the right dlPFC and IFG in empathy potentially top-down modulated the progression of the mirroring activation in the motor system. This recruitment of inhibitory functions likely inhibited the entire sequence of action simulation and contributed, thus, to the MENT recruitment. Accordingly, these data may indicate that empathy first relies upon the internal but only partial simulation of the others' lived experience and, then, inhibition of this simulation. This enables partially disengaging from one's egocentered visuo-spatial referencing system and adopting the other's perspective, on one hand and, on the other hand, representing the lived experience of others *as the others'* experience (Thirioux et al., 2014).

Interestingly, an event-related potentials EEG study investigating pain perception in physicians and matched controls reported an early N110 differentiation between pain and no-pain stimuli over the frontal areas and a late P300 over the centro-parietal regions in controls but not physicians (Decety et al., 2010). These data indicate that physicians down-regulated their empathic response very early toward others' pain, inhibiting the bottom-up processing of pain perception. These early regulation effects would enable freeing up cognitive resources that are indispensable to help patients. These results suggest that physicians have developed specific top-down regulation brain capacities.

## Empathy, sympathy, and burnout in care relationship

### Empathy in care relationship

Two preliminary remarks should be made. First of all, the current growing interest for empathy in medicine contrasts with a form of “detached concern” that has been therefore described in seminal texts from the 1950s as well as the 1960s and has long been considered as the heart in care relationship (Halpern, 2014). In 1906, W. Osler had already defined the neutralization of emotions as the necessary condition for physicians “to see into” their patients and access “their interior life” (Osler, 1963; see Halpern, 2014). According to this approach, the relationship toward patients is intellectualized and excludes any feeling-related dimension. “To know *that*” the patient is in a given mental state is sufficient “to know how” he/she is feeling. Empathy, as multidimensional, complex and integrative phenomenon (“to know how it feels like to”), stands between this neutral and detached concern (“to know that”) and the vicarious emotional sharing (“to feel”) as encountered in sympathy.

Secondly, literature on medical care uses the term of “clinical empathy,” defining, thus, empathy for the patient as a specific category. Contrasting with the divergent definitions of empathy in general (i.e., outside care relationship), the definition of “clinical empathy” benefits from a more precise and consensual conceptualization. Clinical empathy encompasses four dimensions. The feeling-related (or emotional) dimension refers to the capacity to imagine what patients are feeling and experiencing. The cognitive dimension is the higher order capacity to identify and represent the patients' internal experience and viewpoint. The moral dimension concerns the physician's motivation to empathize with the patient. Finally, the behavioral dimension refers to the physician's capability to communicate to the patient that his/her viewpoint and the content of his/her experience have been understood and taken into account (Halpern, 2014). The last two dimensions, necessary to clinical empathy, are proper to the nature of care relationship and ethics of care. However, the visuo-spatial and self-regulation dimensions of empathy in general are not included in its clinical definition. We here believe that taking into account these two main features is important to have a better understanding of the link between burnout and empathy in physicians but also for burnout prevention.

Even though the interest toward clinical empathy has significantly increased since the last decade, there are only little experimental and observational studies on empathy in physicians. The same is also true for sympathy. However, it has been shown that physicians' empathy impacts the quality of both diagnosis and care but also the adherence and efficacy of the prescribed treatment (Kim et al., 2004). The principal qualities that patients expect from their GPs are humanity, notable efforts in understanding their difficulties and good communication skills (Wensing et al., 1998; Cape et al., 2000). Furthermore, patients tend to recommend their GPs if they judge that these are empathic (Vedsted and Heje, 2008). Moreover, it has been demonstrated that patients, before verbally describing private and emotional aspects of their clinical history, give non-verbal cues concerning these clinical elements through bodily postures and specific gestures. Patients have a significantly greater tendency to reveal clinical details if their physician non-verbally responds to these non-verbal cues, compared to verbal answers (Roter et al., 2006). In the same vein, physicians' empathy has been further shown to reinforce the patients' trust and facilitate the description of pertinent symptoms and details which are important for the diagnosis (Roter et al., 2006). Recent data indicate that the physicians' clinical competences, when these are evaluated by an exterior peer by means of an Objective Structured Clinical Examination, positively correlate with their empathic abilities as reflected during the clinical consultation. This correlation is associated with a greater evaluation of the physicians' clinical performance by the patient. In contrast, evaluation by physicians of their own empathic skills did not correlate with their clinical competences (Ogle et al., 2013). Many physicians gain a greater trust from patients that furthermore increases thanks to their high empathic skills, which improves patients' adherence to treatment. These are important results because only 50% of all treatments are taken as prescribed (Halpern, 2014). Concordant with these data, a study involving 891 diabetic patients and 29 physicians revealed that higher scores on the Jefferson Scale of Empathy (measuring clinical empathy) [JSE] in physicians (Hojat et al., 2001) are associated with indicators of diabetic control (hemoglobin A 1c < 7.0% and low-density lipoprotein cholesterol < 100) in patients (Hojat et al., 2013). Another study with 242 physicians and 20,961 diabetic patients demonstrated that greater empathic skills in physicians are associated with a diminution of metabolic complications necessitating hospitalization (Hojat et al., 2013). Moreover, Hojat et al. (2011a) have compared the scores obtained on the JSE and on the Interpersonal Reactive Index (IRI) (Davis, 1983) in medical students and residents. IRI is a four 7-items subscale self-report assessing general empathic abilities and notably its cognitive (i.e., Fantasy Scale and Perspective-taking) and emotional dimensions (i.e., Empathic Concern and Personal Distress). Higher scores on the Perspective-Taking subscale were associated with greater clinical empathy skills. Medical students and residents who obtained higher scores on the Personal Distress subscale, which measures the tendency to sympathize with other rather than empathic traits, had lower JSE scores. This study suggests that the tendency to merge with others and attribute to oneself what others are experiencing as encountered in sympathy increases personal distress. This is specifically true when physicians are confronted with the others' psychological and physical pain and associated with a clinical empathy decrease.

To sum it all up, empathy in medical care benefits both physicians and patients. Firstly, physicians' empathy is associated with greater clinical competences and care efficacy. The more empathic physicians are, the more patients adhere to treatment and understand medical indications. Moreover, physicians' empathy has a positive effect on the patients' quality of life as well as physical, psychological and social well-being. Finally, empathy also positively impacts the physicians' quality of life and well-being: physicians evaluate an empathic relationship to patients as generating a greater professional satisfaction (Halpern, 2014).

### Burnout in care relationship and physicians: confusion between empathy and sympathy?

If empathy is beneficial for physicians and at the root of a greater professional satisfaction, it seems, thus, to be a burnout preventive factor. However, theoretical hypotheses propose that empathy causes burnout. How two theoretical models that try to explain the same phenomenon, i.e., the link between burnout and empathy, may advance an opposite causal relationship between burnout occurrence and empathy? We here do not pretend that the relationship to the other *per se* is the unique factor of burnout in physicians. We believe that burnout has a multifactorial origin but that burnout in physicians is nonetheless specific insofar as care relationship is a specific relation to others. We hypothesize that burnout has a multiple etiopathogeny but that the nature of the care relationship facilitates burnout occurrence. It means that triggering factors, which are respectively independent (e.g., personality traits, environment etc.) and dependent of the care relationship nature, should be distinguished with caution.

According to the Theory of Compassion Fatigue, burnout in physicians is associated with an excessive empathy (Figley, 2002; see also Tei et al., 2014). Physicians with over-exaggerated empathic abilities would have more chances to suffer from emotional exhaustion, leading to compassion fatigue and, then, burnout (Nielsen and Tulinius, 2009). This feeling of exhaustion would be due to the difficulties that physicians encounter with certain patients, i.e., in a care relationship that necessitates sustained listening and attention. C. R. Figeley further proposes that compassion fatigue corresponds to a state of extreme strain and unremitting concern for the patients' pain, leading physicians to feel interiorly traumatized. On the contrary, the Theory of Emotional Dissonance posits that burnout is associated with diminished empathic capacities (Abraham, 1999; Larson and Yao, 2005; Tei et al., 2014). This hypotrophy of empathy would originate in alexithymic tendencies (Gleichgerrcht and Decety, 2013), i.e., difficulties in identifying, differentiating and describing one's own emotions and feelings, in association with impoverished mental representations of one's and others' emotional states. Physicians with alexithymic traits would have, thus, difficulties in representing patients' feelings. According to these two theories, empathy causes burnout. However, these models differ as a hyper-functionality (hypertrophy) of empathy is the direct cause of burnout in the Theory of Compassion Fatigue whereas a hypo-functionality (hypotrophy) of empathy is the indirect cause of burnout in the Theory of Emotional Dissonance. A third theoretical hypothesis describes empathy not as causing but preventing burnout because it generates a greater professional satisfaction (Halpern, 2003). Finally, according to a fourth hypothesis, burnout alters empathy. Depersonalization in burnout would lead to dehumanizing the relationship to patients and objectifying patients, based on defense and protection mechanisms, and to consequently altering empathy (Shanafelt et al., 2005; Brazeau et al., 2010; Zenasni et al., 2012). GPs suffering from depersonalization would be impaired in adopting the psychological perspective of their patients and tend to keep them at distance (Truchot et al., 2011; Zenasni et al., 2012).

**Figure 2:**
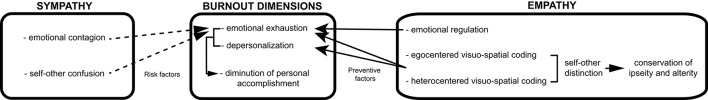
**A process model of the link between burnout, empathy and sympathy in physicians**. Empathy toward patients is a preventive factor of burnout. Emotional regulation prevents emotional exhaustion. Dynamic egocentered and heterocentered coding enables self-other distinction. Maintaining both ipseity and alterity, these empathic processes prevent both emotional exhaustion and depersonalization. In contrast, sympathy toward patients is a risk factor of burnout. The components of emotional contagion and self-other confusion in sympathy causes emotional exhaustion (black and dotted arrows respectively indicate preventive and risk factors of burnout).

We believe that solving these theoretical contradictions requires taking into account (1) the multidimensional nature of burnout, (2) the distinction between empathy and sympathy but also (3) how the different burnout dimensions respectively relate to the different dimensions of empathy and sympathy. For that, we propose a bio-psychopathological approach. We hypothesize that empathy is a preventive factor of burnout. Empathy would prevent burnout occurrence because it not only gives sense to the medical practice and generates a greater professional satisfaction but also puts at stake neuro-functional and neuro-cognitive mechanisms which make a stable, non-pathological and non-pathogenic relationship to others in general and patients in particular possible. Empathy as multidimensional and integrative phenomenon relies upon a stable relation (integrative feature) between different dimensions (multidimensional feature) which should be neither hypo- nor hypertrophied. A genuine empathy relies, thus, upon a stable relation between its emotional and cognitive dimensions. This is made possible by the visuo-spatial and self-regulation processes which maintain self-other distinction and prevent to inappropriately attribute to oneself what others are currently experiencing. The ego-heterocentered dynamic coding and partial inhibition of one's egocentered perspective in empathy avoids merging with others and, thus, a consecutive weakening and loss of the Self. This further enables to keep the other in his/her alterity. We first propose that empathy is a preventive factor of the emotional exhaustion dimension of burnout in physicians (Figure 2, Table 1). This is due to the fact that empathy encompasses emotional self-regulation that enables physicians to feel and understand the patients' emotions but without entirely simulating these emotions or attributing these emotions to themselves. In contrast, sympathizing with patients would cause emotional exhaustion as sympathy relies upon both self-other confusion and self-attribution of what patients are experiencing (Figure 2, Table 1). We further hypothesize that empathy prevents depersonalization. It would be due to that empathy is based upon a clear and distinct consciousness of self-other distinction and therefore enables the consideration of others in their alterity, idiosyncrasy and status as Subject. This process renders the relationship to others immune to any objectification (Figure 2). Our hypothesis is also compatible with the theoretical model positing that depersonalization in burnout would cause an alteration of empathy, suggesting, for this specific dimension, a reciprocal but non-contradictory causal relationship between burnout occurrence and empathy.

Interesting data report that low scores on the IRI Perspective-Taking subscale is a risk factor to develop burnout whereas high scores to the Perspective-Taking and Empathic Concern subscales are protective factors (Lamothe et al., 2014). A recent study with 7584 physicians has shown that compassion satisfaction and compassion fatigue are respectively associated with higher scores on the IRI Perspective-Taking and Personal Distress subscales (Gleichgerrcht and Decety, 2013). Considered collectively, these results seem to corroborate our hypotheses, although this validation is only partial insofar as these studies have not yet tested for potential correlation between the different dimensions of burnout and IRI. Tei et al. (2014) in a recent neuroimaging study have demonstrated that modulations of activations in brain networks sustaining empathy predict burnout severity in physicians. That is, activations in the anterior insula (coding for bodily self-awareness and somaesthetic insight) and TPJ (coding for self-other distinction and perspective change) are significantly decreased in physicians suffering from burnout. Moreover, this diminished activation correlates with burnout severity. Although these data foster a better description of the empathy-burnout link in physicians, new research paradigms need to be developed in order to have a better understanding of the neuro-functional mechanisms sustaining the modality of this link and to validate or invalidate the different existing hypotheses. This is an important issue because understanding these dysfunctions will enable to more efficiently prevent burnout and specifically train more students in their medical education.

## Conclusive considerations and perspectives

The clinical relationship toward patients starts from the third year of medical education in France. Paradoxically, although a daily exposure to the physical and psychological pain of other people has been well-documented to affect healthcare professionals, the impact of such a constant exposure on the health of medical students does not benefit from any sort of validated measurement tool. This is an important issue because observational studies, as mentioned above, report that emotional exhaustion and empathy respectively increases and decreases with the number of years of medical training. Moreover, “patient-physician relationship” and “empathy” are two evaluation items of the French national exam for medical residency. Again, there are no common validated and accepted bases that describe how these two fundamental care relationship components should be theoretically and practically taught. Furthermore, the medical teaching, focusing on both the patient-physicians relationship and empathy has been reported to only have a very low impact on burnout prevention and empathy decrease (Truchot, 2006). It means that there is no common tool that enables (1) assessing whether emotional exhaustion, tendency to sympathize with patients and alteration of empathy effectively correlate and (2) preventing both burnout occurrence and empathy decrease in medical students. There is also no common tool enabling to theoretically and practically teach medical students about how to protect themselves from burnout as well as develop and/or maintain their empathic skills.

Here, we propose to develop specific training programs that may potentially contribute to the prevention of burnout in medical students as well as senior physicians. Concerning medical education, we firstly propose to systematically track medical students who resist and those who do not resist burnout. We hypothesize that the former students have respectively high and low scores to the IRI Perspective-taking and Personal Distress subscales whereas the later would report an inverse pattern. Secondly, we propose to develop a “practice of care relationship” based upon two main pedagogical tools and therapy-based methods. These would enable medical students to mentally adopt the patients' perspective while actively maintaining self-other distinction and reinforcing their emotional self-regulation mechanisms. That is, the Mindfulness Method, an embodied cognition approach which positively impacts burnout, and the Analysis of Anxiety Situations Method, a reflexive approach which is used in behavioral and cognitive therapies. This practice of care relationship would be taught in the initial medical training but also in the continuous professional formation, targeting senior physicians who suffer from burnout or report exaggerated tendencies to sympathize with patients. Our aim in developing this program is to enable students and physicians to have a better control of their relationship toward patients and, thus, to potentially reduce the deleterious burnout consequences on physicians' health and, thus, medical errors. However, it is worth iterating that burnout has a multifactorial origin. Therefore, our empathy-based training programs would only focus on one of the incriminated potential factors of burnout. It means that such programs could have limited impact if the other personal, developmental-psychodynamic, professional and environmental factors are not also taken into account and evaluated in parallel. We believe that giving a better understanding of burnout in physicians as “pathology of care relationship” and how empathy- and sympathy-like relationships toward patients may respectively preserve and cause burnout could not be envisaged without considering the empathy-sympathy-factor within the multifactorial nature of burnout.

## Author contributions

BT, FB, and NJ chose the topic. BT conceived the design of the manuscript and wrote the main text. FB was in charge of the Sections Burnout in Care Relationship and Conclusive Considerations and Perspectives. NJ revised the manuscript. All the authors checked and approved the final submitted version of the manuscript.

### Conflict of interest statement

The authors declare that the research was conducted in the absence of any commercial or financial relationships that could be construed as a potential conflict of interest.
